# Challenges with seeking HIV care services: perspectives of older adults infected with HIV in western Kenya

**DOI:** 10.1186/s12889-019-7283-2

**Published:** 2019-07-11

**Authors:** Jepchirchir Kiplagat, Ann Mwangi, Charles Chasela, Susann Huschke

**Affiliations:** 1 College of Health Sciences, School of Medicine, Moi University, Eldoret, Kenya; 2 Academic Model Providing Access to Healthcare, Eldoret, Kenya; 3 Faculty of Health Sciences, School of Public Health, University of the Witwatersrand, Johannesburg, South Africa; 4 Right to Care, EQUIP, 1006 Lenchen North Avenue, Centurion, Pretoria, South Africa; 5 Graduate Entry Medical School, University of Limerick, Limerick, Ireland

**Keywords:** Older adults, HIV, Engagement in care, Experiences, Comorbidities, Challenges, Kenya

## Abstract

**Background:**

While younger adults (15–49 years) form the majority of the population living with HIV, older adults (≥50 years) infected with HIV face multiple challenges related to the aging process and HIV. We explored the experiences of older persons infected with HIV at the Academic Model Providing Access to Healthcare (AMPATH) program in western Kenya to understand the challenges faced when seeking HIV care services.

**Methods:**

Between November 2016 and April 2017, a total of 57 adults aged 50 years and above were recruited from two AMPATH facilities – one rural and one urban facility. A total of 25 in-depth interviews and four focus group discussions were conducted, audio-recorded, transcribed and thematic analysis performed.

**Results:**

Study participants raised unique challenges with seeking HIV care that include visits to multiple healthcare providers to manage HIV and comorbidities and as a result impact on their adherence to medication and clinical visits. Challenges with inadequate quality of facilities and poor patient-provider communication were also raised. Participants’ preference for matched gender and older age for care providers that serve older patients were identified.

**Conclusion:**

Results indicate multiple challenges faced by older adults that need attention in ensuring continuous engagement in HIV care. Targeted HIV care for older adults would, therefore, significantly improve their access to and experience of HIV care. Of key importance is the integration of other chronic diseases into HIV care and employing staff that matches the needs of older adults.

**Electronic supplementary material:**

The online version of this article (10.1186/s12889-019-7283-2) contains supplementary material, which is available to authorized users.

## Background

Older adults living with human immunodeficiency virus (HIV) continue to increase in number owing to wide access of antiretroviral treatment (ART) that has resulted in living longer with HIV and new HIV diagnoses among persons aged 50 years and above [1]. Sub-Sahara Africa bears the greatest burden of HIV and hosts over 74% of all older adults living with HIV. In Kenya, the prevalence (5.6%) [2] of HIV among older adults is estimated to be similar to that of the general population. A recent analysis from data in western Kenya estimates that older adults account for 12% of all adults living with HIV [3]. While younger adults (15–49 years) form the majority of the population living with HIV, older adults (≥50 years) infected with HIV face multiple challenges related to the aging process.

As the HIV infected population ages, they are susceptible to developing other chronic illnesses such as cardiovascular diseases, chronic obstructive lung diseases, diabetes, declining liver and kidney functions [4], osteoporosis, arthritis, cancers and mental health conditions [5]. These illnesses pose a challenge to both the HIV infected individual and the healthcare provider. The timing of antiretroviral treatment (ART) initiation is affected by the presence of comorbidities in older adults [6, 7], as these may necessitate a delay in initiation. If ART is initiated in the presence of co-morbidities, care providers have to closely monitor ART regimens for early switching in cases of ART toxicities [8, 9]. Furthermore, HIV patients with comorbid conditions, require multiple prescriptions, which not only make it difficult to track the medication but also threaten patients’ ability to adhere to ART [10]. Previous research has shown that older adults who are nonadherent to ART are more likely to present with depressive symptoms associated with stigma and loneliness [11].

More than three decades into the HIV epidemic, stigma and discrimination towards persons infected with HIV continue to persist. Older persons living with HIV experience stigma related to HIV [12] and discrimination due to age [13]. In some cases, older persons have been branded too old to be HIV infected [13, 14]. Stigma and discrimination have, therefore, become a barrier to accessing HIV services [15] including testing and care services. Additionally, stigma directed towards persons infected with HIV has made it difficult to cope with their illness [16] and has been associated with poor health outcomes among the older population [12]. Some HIV infected older persons have shied away from disclosing their status for fear of being stigmatized [12, 17, 18], which in turn prevents them from receiving social support from those close to them.

Social support is a significant resource for aging persons, especially those suffering from chronic conditions [19], including HIV. Social support is positively associated with the psychosocial wellbeing of a person. HIV is a socially challenging condition because of its association with sexual and stigmatized behaviors [20]. Those infected, particularly older people, often live a socially isolated life [21, 22], with less social support, resulting in negative health outcomes, including morbidity and mortality [23]. It is therefore evident that older adults have unique needs for healthcare that differ from those of younger adults, though limited research has been done to examine older adults’ experiences in utilizing HIV care services.

Qualitative research related to older adults in Kenya has generally looked at the impact of HIV on older adults as caregivers. There is little information available on HIV infected older persons’ experiences with seeking HIV care services. Yet older adults continue to be at risk of HIV infection and death from AIDS. With this information gap, we conducted in-depth interviews and focus group discussions to explore the challenges faced by older adults living with HIV in accessing and engaging in HIV care services at the AMPATH program in western Kenya.

## Methods

### Study setting

The study was conducted at the Academic Model Providing Access to Healthcare (AMPATH) program. The program is a joint partnership between Moi University, Moi Teaching and Referral Hospital (MTRH) and Indiana University School of Medicine. The AMPATH program provides comprehensive HIV care serving a population of over 4 million people in western Kenya. AMPATH is the largest program providing HIV care and treatment for free in Kenya and has enrolled over 200,000 HIV infected patients with over 90,000 of them currently on ART [24] [25].

In the last decade, the AMPATH program has evolved to include primary healthcare (PHC) services and chronic disease management (CDM). HIV care services are provided within the comprehensive care clinics (CCCs) while PHC and CDM are provided within the same facility but by different care providers requiring patients to be referred from one provider to another depending on the conditions they are suffering from, other than HIV.

Within the care facilities, patients are provided with patient identifiers (IDs). One patient is likely to have multiple patient IDs depending on the care services sought and received. For example, all persons infected with HIV are provided with unique AMPATH medical record system (AMRS) identification and if a patient is referred for diabetes or hypertension care, they will be provided with a different identification. Similarly, if they visit the cancer clinics or mental health clinic, they will obtain IDs different from those of other clinics visited. All of these identifications are managed separately. Hence, the patients are managed independently of any other condition they could be suffering from. Additionally, provision of care for specialized conditions is offered on specific days of the week, at times requiring patients to make multiple visits to the facility for different conditions. Patients visiting rural facilities may require referrals to higher level facilities, often situated in urban areas, for specialized care services that include cancer care and management.

HIV treatment in Kenya and at AMPATH are offered to all patients at no cost. However, persons infected with HIV requiring primary care services, care for opportunistic diseases (except for tuberculosis treatment) or care for other chronic diseases are expected to cover the costs associated with these other services including medication costs.

### Study design

This study is part of a larger mixed methods study looking at characteristics, outcomes, and experiences of older adults living with HIV. The descriptive qualitative study we report in this article was conducted between November 2016 and March 2017. We adopted the qualitative approach to gain more understanding of the experiences and the challenges older adults face when seeking HIV care services. We focused on understanding the challenges older people face after noting that older adults were more likely than younger adults to be diagnosed late into their HIV infection [3], and this was likely as a result of HIV testing services not targeting this population [26].

Study participants were purposively recruited from two HIV care facilities of AMPATH, Mosoriot (rural) and MTRH (urban) sites. In-depth interviews and focus group discussions methods of data collection were employed. A socio-ecological model [27] previously used to assess the facilitators and barriers that impact retention in HIV care was adopted in this study. We used the model to explore challenges experienced by older adults while engaging in HIV care services at various levels that include intrapersonal, relational, community and organizational levels.

### Target population

We targeted all HIV infected persons who were aged 50 years and above at enrolment into HIV care facilities and were currently receiving care at the two facilities of the AMPATH program. We adopted the older persons’ WHO definition [28] for those infected with HIV. Older persons enrolled at the MTRH and Mosoriot clinic sites were recruited. Participants aged ≥50 years at enrolment in HIV care, were receiving care at Mosoriot and MTRH clinics with a one year follow-up period were included in our study.

### Data collection

Training on data collection tools – IDI tool (Additional file 1) and FGD tool (Additional file 2) and data collection process was done for the two research assistants (RAs), female aged 52 years and male aged 55 years. The male and the female Ras interviewed male and female participants respectively. The tools were pilot-tested and questions refined based on the responses received. The tools were then translated to Kiswahili, a language that was used during the interviews.

#### Participant identification

A detailed description of how participants in this study were identified is provided in a different but related paper [26]. In brief, RAs extracted a list of persons aged 50 years and above from the list of clinic attendees of the day. Age at the time of engaging in care was noted. Clinicians who were already aware of the study were requested to inform the Ras when a potential participant completes a clinic visit. The RA approached potential participants and provided an explanation and the purpose of the study. Those participants who agreed to participate, were invited to choose a convenient interview date and time. Participant’s comfort and privacy were considered during the interview. Of the 25 participants who agreed to participate, one participant requested to be interviewed at a later date. Details of the one participant were requested for the purpose of scheduling the interview meeting. FGD participants were also identified from the clinic with similar characteristics to those of IDIs. Contact details were obtained for further communication with participants on the interview venue, date and time.

#### Interview process

During the in-depth interviews, the purpose of the study was explained including audio-recording of the sessions and written informed consent obtained from each participant. A total of eight (8) participants indicated their consent to participation using a thumbprint. Participants were invited to have someone they trusted to sit with them in the interview, but none of them wanted a companion. The participants’ demographic information was recorded on the interview notes prior to turning the audio recorder on. The session began with a question gauging participants’ understanding of HIV. Participants were then invited to narrate their experiences living with HIV, experiences during testing, linkage to care, treatment and retention in care, disclosure, stigma and discrimination, substance and alcohol use, adherence to clinic and medication, type of social support, and healthcare needs and satisfaction with care services. After the interview, each participant received 500 Kenya shillings (USD 5) as reimbursement for the time spent during the interviews. IDIs lasted between 40 min and 115 min.

A total of four FGDs were conducted, two in the rural facility – one for female and one for male and the other two in the urban facility – one constituting male and the another for female. Similar questions to those asked in IDIs were asked during FGDs. The FGD interviews were held in the medical training college near the health facility for the rural group while the urban FGDs were conducted within the teaching hospital. Interviews were held for female and male separately. The room was set up, so the participants sat on an oval-shaped table with the facilitator and note-taker sitting among the participants. An audio recorder was placed in the middle of the table to ensure good coverage of the recording. Prior to beginning the interviews, the facilitator explained the purpose of the study including audio recording procedures and sought consent from participants. Oral consent was obtained from FGD participants. Refreshments were served and transport reimbursement of USD 5 was provided to participants after the FGDs were completed. FGDs took between 90 min and 135 min.

### Data management and analysis

Audio-recorded data from IDIs and FGDs were transcribed verbatim and translated into English. For coding purposes, we uploaded all the transcripts to NVIVO version 10. Thematic analysis [29] was conducted and involved the identification of themes using inductive methods and deductive using overarching concepts (intrapersonal, interpersonal, organizational, and community levels) in the socio-ecological model. The first author read several transcripts and inductively developed a codebook based on emerging patterns. The codebook was then reviewed by three qualitative experts. Their input was incorporated in the final codebook and was used to analyze all the transcripts. While the concepts in the socio-ecological model (individual-level, community-level, facility-level, and policy-level factors) guided the analysis, the authors noted that several factors interacted at various levels and influenced each other in many ways. After reaching a consensus among all authors the codes were then grouped around three main themes: i) lack of resources, ii) clinic facilities and staff, and iii) social responsibilities. We searched for associations of concepts to any of the three main themes and interpreted the results. This study also followed a rigorous process as recommended for qualitative research [30]. The findings of this study have been summarized according to the themes and subthemes that emerged.

## Results

### Participants’ characteristics

Participants’ characteristics have been described elsewhere [26]. In brief, 57 participants, (25 IDI participants and 32 FGD participants), with their ages ranging from 54 to 79 years were recruited in our study. Of the total participants interviewed, 30 (53%) of them were male and 27 (47%) were female and more than half (57%) had obtained a formal education. Thirty-one, (54%) of the participants were attending an urban clinic and the remaining participants were seeking care at a rural clinic. At the time of the interviews, slightly above half (54%) were married and living with their spouse while the remaining were either widowed, separated or divorced.

In the sections below, we present experiences of older adults infected with HIV and summarize the challenges faced when seeking HIV care services. All participants in our study were engaging in care at the time of interviews and the majority of them had regularly engaged in care. Despite this, participants reported challenges in engaging in care including difficulties in managing HIV and comorbidities (individual-level and facility-level factors), unmatched age and gender of healthcare providers (facility-level factor), poor patient-provider communication (facility-level factor), poor quality of healthcare facilities (facility-level factor), stigma and discrimination (community-level and facility-level factors), inadequate social support that includes transport issues (individual-level and policy-level factors), and caregiving responsibilities (individual-level and community level factors).

### Lack of resources

#### HIV and other comorbid conditions

Of the 57 participants interviewed in this study, 13 participants stated that they were suffering from one or more chronic conditions other than HIV. The conditions mentioned included arthritis, diabetes, cancer, hypertension, chronic kidney disease, and liver diseases. Participants described these conditions as needing urgent care, more so than HIV. Some of the conditions, such as hypertension and cancer were described as critical, as they can result in sudden death. These descriptions suggested prioritization of the management of comorbid conditions as they were considered more critical compared to HIV. Additionally, participants shared frustrations in the lack of patient-centered and coordinated scheduling for patients who sought care from multiple providers. Time taken, and costs incurred in seeking medical services drained the limited resources available within the family.

#### One of the participants described her experience living with HIV, hypertension, and diabetes

*After about a year or two of diagnosis with HIV, my doctor told me that my sugars were high and I needed to see another doctor. (…) I went to the clinic, and I was told to come the next day since the diabetes clinic was closed that day. I went home stressed because my sister had died of diabetes after suffering from daily injections for a long time. I came back the day after and I was asked to go back home and come again very early in the morning without taking breakfast. I came and they took the test and was told that I had diabetes. I was given medication and asked to come after a month (…). The doctor told me that my sugars were still high and I was sent to a nutritionist who wrote the type of food I should eat. (…) It was not easy for me. The medicine for sugar was very expensive, and I did not have money. I would sometimes borrow because my children are not well off. They are also struggling with life. Two months ago, the doctor told me that my ‘heart is beating a lot’ [local description of hypertension]. Today I was coming for the heart clinic, then I will come again next week for the diabetes clinic. It is really costly (…). The medicine for HIV is the only one that is free. I buy the others (…) sometimes I even miss to come for the HIV clinic because I use all the money to buy medicine for diabetes and hypertension [with no money left for transport].*

IDI, female, urban.

Another participant suffering from cancer was admitted to the hospital and missed her ART medication described her experience living with multiple chronic conditions.

*I also have cervical cancer that was diagnosed last year. I have been on chemotherapy and it is really painful. (..) I was admitted for three months [at the hospital] and I missed ARVs for two months. They say I could not even talk (…) that I was dead, but God saved me.*

IDI, female, rural.

These quotes indicate that the time, effort and money required to attend to co-morbidities such as diabetes and cancer (individual-level factor) can prevent older patients from accessing HIV treatment and managing their ART. Additionally, the lack of integration of care services (facility-level factor) for HIV and other chronic diseases have a negative impact on continuous engagement in care.

#### Caregiving responsibilities

Most of the participants mentioned that they had other responsibilities in addition to taking care of themselves and their own health. They had children, grandchildren, farms, and animals that they needed to look after. Faced with financial challenges and prevalent poverty levels, participants described the struggle of often choosing between using the small amount of money they have to pay school fees and buy food – with a good diet considered particularly important for those living with HIV - or pay for transportation to the health facility. In cases where there was no help available, some of the participants would spend time grazing their animals or tending to plants on the farm instead of visiting the healthcare facility for their HIV care. More women described caregiving responsibilities than their male counterparts during the interviews.

*I took care of my daughter who died of HIV. She left behind 3 children. One of them is HIV infected. I always bring her when coming for the clinic. She just went home with my neighbor so I can attend this meeting. It is not easy taking care of grandchildren needing a lot of attention and medication every day, and being on medicine myself. I sometimes have to choose between buying porridge or use the money for transport to the clinic. You know it is far and we are not able to walk here and back (…). So we both miss the clinic sometimes.*

FGD, female, urban.

Another participant who has to take care of his farm and animals in order to educate his children described his experience with attending clinic visits and adherence to medication.

*I am now paying school fees for my children, two are in secondary and another one is doing his Kenya certificate of primary education this year. I depend on farming, selling milk and maize from the farm. Since my wife is unwell, I have to take the cows for grazing. A times, I miss the lunchtime medicine because I may not be back on time to take it at home. (…) I try so much to come to the clinic but I must confess that I have missed the clinic because I had to work on the farm. (…) When you explain this to the doctors, they don’t really understand.*

IDI, male, rural.

The above quotes exemplify the interaction between various level factors that deter or facilitate older adults’ care seeking. Poverty levels and food insecurity (policy-level factors) forces older adults to forego their own health in order to provide to their children and grandchildren. As a result, older adults report missing clinic visits (facility-level) and poor adherence to medication (individual-level).

#### Distance to the clinic

Older adults described the struggles they face with costs of transportation when accessing the facilities to seek HIV care services. They shared their experiences in trying to find money, at times doing casual work like weeding or washing clothes for others to get money for transport. Some participants suggested that more clinics should be opened in rural areas to reduce the distance to be traveled, or that healthcare providers should distribute medication to patients in the community instead of asking them to travel to the clinic.

*No one gives me any money. I have to work for people to get money to pay for transport to come to the clinic. I sold most of my land to cover the bill when my wife died after six months in the hospital and now the little I have, I cannot rent it out. I try and plant vegetables and sell, but it is difficult. I wish they could open a clinic at the dispensary because we can walk there.*

IDI, male, rural.

*There is a time I had to walk all the way to the hospital. It took me three hours. My feet were starting to swell and were painful. When I got here, I told the doctor I did not have transport, and he gave me 50 shillings [~US$ 0.5]. I was so grateful because I did not know how I would get back (…). I would really request the management to have doctors visit old people in their homes and provide them with medication to support those who are not able to come to the clinic. I have seen it done for TB patients and I think they can do that for us, too.*

FGD, female, rural.

Long distances to the care facilities (facility-level factor) impacted participants’ adherence to clinic appointments due to lack of transportation. As a result, participants sought employment (individual-level factor) to get money to facilitate their travel to clinics with others, despite the fragility (individual-level factor) opting to walk the long distances.

### Clinic facilities and staff

#### Quality of healthcare facilities

Participants expressed that the quality of the healthcare facilities was not sufficient in many cases, particularly considering that the average waiting time at both clinics (rural and urban) is about three hours. Often during these waiting times, participants indicated requiring frequent visits to the bathrooms. Participants from both urban and rural facilities appreciated the presence of the toilets facilities at the clinics. However, they noted the toilets were often locked or very dirty. Only female participants expressed difficulties in accessing the toilets services at the healthcare facility.

*As older women, you feel the urge to relieve yourself. But the toilets are so dirty that I fear even going inside. I usually go to the maternity ones but now they are closed because of a strike by the nurses. Now I have to really stay pressed until I leave the hospital to find a place to go and relieve myself.*

FGD, female, urban.

Another participant who appreciated the availability of drinking water was concerned that no cups are availed to patients to drink the water when swallowing medication. She described the challenge below.

*I also want to talk about water. If it is possible to provide cups so that we can use to drink water. Sometimes we sit on the queues and feel so thirsty but there are no cups to drink water from the tap. You are forced to use the hands even though they are not clean. So if we could get cups for drinking water that would be good (…) also for swallowing the drugs, there are so many, you cannot swallow them with saliva.*

FGD, female, urban.

Participants were concerned with facility-level factors that impacted their continuous engagement to care. Because of age (individual level factor), older adults develop urine incontinence that requires frequent visits to the toilets. Additionally, participants with comorbid conditions (individual-level factor) like diabetes require frequent water intake or for some for swallowing medication.

#### Age and gender of healthcare provider

During both IDIs and FGDs, participants highlighted the importance of age and gender of the healthcare provider in care provision for older adults. Younger healthcare providers were less likely to discuss sexuality with older adults as this was viewed as a taboo.

*If we had a doctor like Rachel (not her real name) dedicated to only seeing older women we would be very happy. Just like you (referring to the interviewer), she is our agemate and when you talk to her she is very understanding. I can even confide in her on issues related to sex. The problem now is that she sees too many patients and does not have much time to spend on one person because people will complain outside that you have overstayed.*

FGD, Female, urban.

While the age of the care provider was not cited as an issue by male participants, the gender of the healthcare provider was mentioned as an important factor in discussing sex-related issues.

*Sometimes you can have a pressing problem that you want to discuss with the doctor but when you go to the doctor’s room you find a lady doctor. One day I came to the clinic, and I wanted to talk to the doctor about the HIV medicine, if it reduces someone’s sexual activity but I found Dr. Jane (not her real name). I did not tell her about it, but I later looked for Dr. John (not his real name) and he really counseled me. He even prescribed some drugs for me (….) I was almost considered useless in my own house.*

IDI, Male, rural.

These quotes indicate that older adults face challenges discussing sexuality issues that would likely be mitigated by dedicating older and gender-matched healthcare providers (facility-level factor) for HIV infected older adults.

#### Patient-provider communication

Participants applauded healthcare providers whom they described as being friendly and encouraging. Some health care providers who handled older adults gently were described as being respectful and loving. Participants also felt secure and free to speak around such providers.

*This place has really helped us. (…) There is no place like here, there isn’t. (…) The doctors here are doing a very important thing otherwise many people would have died. (…) They are very encouraging and ask us to take our medication. Some other doctors even pray with us and ask God to heal us as they provide medicines.*

IDI, Male, urban.

*I am just grateful, I give them [healthcare providers] a thumbs up. The sisters and doctors treat us very well. Especially older patients. They treat us with respect. They don’t quarrel with us like they do with young people. They even ask us to speak to the young people so they know how to keep to their clinic visits and take their medication well. When I have an issue, I talk to the doctors freely without any fear. You know they joke with us ‘gogos’ [grandmothers] and tell us we will still see the great-great-grand-children.*

IDI, Female, rural.

*They [healthcare providers] talk to me nicely, if I have a problem I tell them and they give me advice. They encourage me to take my asthma and [blood] pressure drugs too in addition to the ARVs.*

IDI, female, rural.

Despite these positive experiences, some participants intimated that during clinic days, they would like to spend more time with healthcare providers and explain what is going on in their lives. Participants felt that the clinicians were overwhelmed by the number of patients needed to be seen in the day and therefore spend limited time with patients, which particularly affects the older adults who may need more time to explain themselves.

*Sometimes the doctors disappear. I think they go for a break. When they come back they find a long queue waiting (…). The queue is always long every time I come. When you get in to see the doctor, he asks questions about your health and if you have been taking your medicine. If you answer the questions, he tells you that he has added more medicine for the next three months. You know, I may want to tell him about some of the things I feel but because he is in a hurry, I don’t feel comfortable telling him. So I tell myself I will explain the next time, but every time the doctors are in a hurry. Maybe it is because there are many people waiting in the line to be seen (…). Maybe getting more doctors to see patients would give us more time to speak with the doctor.*

IDI, male, urban.

These quotes indicate that healthcare providers attitudes and handling of older adults at the clinic (facility-level factor) affect continuous engagement to HIV care.

### Social relationships

#### Social support

Family support was highly commended by older adults in the process of accessing and continuing HIV treatment. Cited by many participants was the importance of having a family member they trust who can offer support when living with HIV. Partner support and support from children and grandchildren was highly rated as a facilitator for adhering to medication and clinic visits.

*My wife has been very supportive and reminds me to take the medication, and even when I travel she calls to check if I have swallowed the medicine. My children send me money to use for transportation to the clinic. They know that I am sick and they are really taking care of me.*

FGD, male, urban.

*I live with my grandchildren. They know grandma takes medication at specific times of the day. When it is time, they run to my room in the morning and wake me up to take medicine. If I have gone to the farm, when they come for lunch from school, they will come to the farm to remind me of my medication. These children are very good. I really love them and they love their grandma too.*

FGD, female, rural.

*My children call me every day to check on my health. They have even taken turns, so one calls in the morning and another in the evening to check if I have taken the medicine. If it was not for them, sometimes I would forget. You know, you wake up and prepare tea and before you know, you have left the house to do other things and can miss taking the drugs. I really thank God for my children. They take care of me.*

IDI, female, urban.

Participants living in discordant relationships shared gendered experiences. Female participants described negative treatment from their non-infected partners that included violence and displacement from matrimonial home while male participants provided descriptions of supportive partners.

*My wife and I were tested. I was positive and she was negative. She vowed to stay with me even if I had messed up. She has really supported me. She ensures that I eat well and that I take my medication on time. We have not disclosed to our children and she is the one who said we should not. If it was not for her, I don’t know how my life would be now.*

IDI, male, rural.

A female participant in a discordant relationship described her experience after testing positive. She was forced to leave her matrimonial home to stay at her aged parents’ home where access to HIV care and treatment was a challenge. She has had to depend on one of her brothers to support her with transportation costs in order to access HIV services.

*My husband and I got tested when counselors came to our house. My test was positive and my husband was negative. He did not say anything after the test, but that evening when he came back, he turned into an animal. In front of our children, he beat me up and insulted me. He threw all my clothes out of our house. I slept at the neighbor’s that day. (…) He was so hostile. (…) I had to go back to my parents’ home. My mother who was then 80 years welcomed me, but things got worse when she passed on. My brothers asked me to go back to my husband, but I could not. Though the others want me to leave, one of my brothers has been supportive.*

FGD, female, rural.

Some participants indicated experiencing memory loss (individual-level) and benefits from family members support (community-level factor) in medication adherence and keeping to clinic visits (individual-level and facility-level factors). Additionally, issues of discordancy that result in gendered experiences (Individual-level and community-level factor) affect continuous engagement in care among older adults.

#### Stigma

Participants interviewed both during the IDIs and in the FGDs mentioned stigma as a major barrier to seeking HIV testing and treatments services. Participants cited cases where if one is seen to visit HIV care facilities, it would be known by the community within a few days. People would begin shunning away from them and would get worse when they disclose their HIV positive status: families might reject them and their community might ostracize them as well.

Participants alluded to cases where they have missed medication for fear of family members seeing the medicine or being seen by a neighbor at the HIV care clinics.

*There was this day I was going for my appointment and I saw my neighbor at a primary care facility. I pretended I was seeking services for malaria and queued to see the primary care nurse. When my neighbor finished with his visit, he waited for me and we walked back home together. That day I went back home without visiting the HIV clinic (…). If he had seen me at HIV clinic, the whole village would have known I am HIV infected*.

IDI, Male, rural.

Another female participant who had shown signs of HIV infection describes her experience with neighbors. She sometimes had to stay and not leave her house for fear of being discriminated against.

*It was really bad. I was so thin and my skin had turned black with rashes. (…) sometimes I could be walking on the road and could see a friend or neighbor approaching. If they see it’s me, they take another route or turn back like they forgot something to avoid meeting or shaking hands. For a very long time, I could just stay in my compound, I did not go anywhere or visit anyone. Even now, there are people who still don’t come to my house.*

FGD, Female, urban.

One way of counteracting stigma is peer support groups for people living with HIV, as facilitated by the AMPATH program. There was some indication that older adults would benefit from peer support groups specifically for the older population living with HIV. Participants who were members of peer groups at the AMPATH program described the sessions as generally helpful because they provided a space to talk to other people in a safe environment, and thereby counteract the stigma and isolation experienced by older people living with HIV. However, older adults reported that they felt out of place as most of the participants were young and suggested that age-specific peer support groups would be more useful.

*I have attended the AMPATH support group twice. We all share our challenges and encourage each other on how to live positively. Some of the people have lived with the virus for more than 20 years and they encourage us a lot. (…) Most of those who attend the support group are young people and if you are old, you feel odd and do not contribute much. If we could start [a group] for older people, that would be really nice.*

FGD, Male, urban.

Stigma and discrimination (community level factor) against those living with HIV is reflected in these quotes and results in non-disclosure of HIV positive status and poor adherence to ART medication (individual-level factors). Peer support groups (community-level factor) are mentioned as a way of counteracting stigma, and specific groups for older adults would be welcomed.

## Discussion

Our study explored the challenges faced by older adults living with HIV in accessing and engaging in HIV care services at the AMPATH program in western Kenya. Our study highlights complex challenges faced by older adults that included difficulty in managing comorbidities in addition to HIV, inadequate facilities, inadequate social support, stigma and poor patient-provider communication. These challenges prevented older adults from the continuous seeking of utilizing HIV care services consistently. As efforts are geared towards achieving the UNAIDS 90–90-90 targets, provision of HIV care should not only focus on HIV treatment but taken into account the aging population and the challenges they face when managing HIV and aging process in their lives [31–36].

Aging predisposes one to comorbid conditions, which are more common among older persons living with HIV [37, 38] compared to younger adults. Our study highlights the challenge of non-coordinated healthcare services for patients suffering from multiple conditions. The cost of seeking care for multiple conditions, often offered by different healthcare providers and likely on different days was not only expressed as being expensive but also hinders continuous HIV care seeking. Our findings concur with previous studies [36, 38–42] conducted in developing countries including studies done by researchers in Uganda’s MRC/UVRI that indicate continuous struggle faced by older adults managing HIV and other chronic diseases. Distance to the facility, cost of traveling to access care, and time [36, 42] have all been cited as barriers to accessing HIV care services among HIV infected older adults. These barriers have been exacerbated by persistent verticalization [42, 43] of HIV care provision. The only comorbid condition that has been fully integrated into HIV care is TB which affects both younger and older adults. Providing integrated care that includes concurrent management of comorbid conditions (e.g. hypertension, diabetes, chronic liver and kidney diseases, mental illness) and HIV, monitoring of drug to drug reactions or interaction and adherence support is, therefore, the key to long-term care for older adults infected with HIV. Previous studies have proposed consideration of comorbidities in the provision of optimal treatment that is patient-centered to older HIV infected persons [35, 36, 38, 44]. Integrating chronic diseases in HIV management can significantly cut down time and money costs and therefore, enhance continuing engagement in care for older persons. Besides, it might address the issue of stigma – where care is provided for all HIV-infected and non-infected by one care provider.

Findings from our study indicate that several years into the HIV epidemic, stigma is still a challenge and more so to older adults, confirming findings from other studies [12, 13, 45, 46]. Compared to younger adults, older adults living with HIV face double stigma – the stigma attached to HIV infections, and the stigma attached to engaging in sex at an older age. The stigma attached to living with HIV continues to prevent infected persons from accessing HIV care services [35]. Public awareness, through campaigns, is still needed to sensitize the community to reduce the stigma associated with HIV among older persons. These campaigns should include the experiences of older adults aimed at challenging the myth that older adults are not vulnerable to sexually transmitted infections (STIs). Once the public is accustomed to HIV infection among older adults, it might lead to increased support for them within the community and in healthcare facilities.

Participants in our study identified social support for persons living with HIV as an important factor in developing a supportive connection and improving HIV treatment adherence. Previous studies, including work done by researchers in Uganda Virus Research Institute, have also shown that social support improves HIV coping strategies [34, 47, 48], promote self-management of chronic diseases [10] and reduce experiences with stigma and discrimination [40, 47]. Unlike younger adults who have a circle of friends and would likely receive social support from their network [49], older adults are more likely have either lost ties with their friends or are widowed and are not quick to form new relationships [21]. We also found in our study that women are more supportive of their husbands when they are tested positive while the opposite may not always be true. The question ‘how do we get men to care about their discordant partners?’ need to be explored to promote partner support in discordant relationships.

Initiating ART for HIV-infected persons is key to survival. Older adults living with HIV and on ART, therefore, require continuous engagement in HIV care services, often facilitated by friendly services [50]. Findings from our study concur with previous studies that quality patient-provider communication is key to HIV care provision [51, 52]. Additionally, older adults seeking HIV care indicated preferences for older care providers and of similar gender. Though previous studies have highlighted difficulties in discussing sexual issues with older adults [53–55] our study sheds more light on how to mitigate this barrier. Our study participants indicated being more likely to ‘open up’ and discuss sexual-related issues including erectile dysfunction, the urge to continue having sex and use of condom at old age with care providers that are of similar age and gender. Our study, therefore, suggests deploying older healthcare providers to serve older patients in HIV care facilities. Same-sex/age support groups may also be useful to reinforce positive behavior.

Lastly, our study found that while older adults have been largely described as caregivers to persons infected with HIV and orphans [56], this responsibility has extended to include taking care of oneself and managing HIV in their lives. Our study demonstrates that while caregiving responsibilities are burdening to older adults, support by grandchildren can also improve adherence to medication. However, participants in our study continued to struggle with prioritizing providing a balanced diet, clothing, and education to children over their own health and wellbeing. Provision of social protection mechanisms [40, 57] will, therefore, go a long way in ensuring older adults cope with the financial burden they bear and improve continuous HIV care seeking.

Whereas the factors discussed above would have been described within the specific concepts (individual-level, community-level, facility-level, and policy-level) of the socio-ecological model, we found that these factors interact with each other and transcend the individual, community, facility and policy levels. We, therefore, suggest that interventions targeting only one specific level might not achieve maximum benefits. Instead, HIV care services for older adults should adopt an integrated, holistic approach that addresses these intersecting factors across various levels.

While this study has highlighted challenges faced by older adults in seeking and receiving HIV care services, it is not without limitations. The persons interviewed in this study were patients that are currently engaged in HIV care. We did not attempt to contact those who had been lost to follow up (LTFU) or those disengaged in care. We believe patients who were LTFU may have more negative or different opinions from those we present in our paper. Since we choose older persons who had been in care for at least a year, we believe their experiences may have improved over time in their survival with HIV and affected their utilization of HIV care services and may not represent experiences of those that are newly infected. However, our findings do raise a number of important issues related to the experiences of older people in accessing and continuing with HIV care that are worth taking into account in improving care for older persons.

## Conclusion

Participants in this study raised several challenges faced when seeking HIV care services including difficulty in managing comorbidities in addition to HIV, inadequate quality of facilities, and poor patient-provider communication. We suggest that targeted HIV care for older adults would significantly improve their access to and experience of HIV care. This tailored service should integrate treatment for other chronic diseases, deploy clinicians or train community health workers that match the age and gender of participants and ensure that patients have access to clean toilets, cups, and water. Furthermore, financial and nutritional support for older people in need would be beneficial. Providing HIV in the community – as near as possible to the older adults – would help ensure that older adults can access HIV care and adhere to their ARV treatments. Last but not least, the stigma attached to living with HIV continues to disrupt people’s attempts to receive treatment and needs to be challenged through public health and awareness campaigns. These should include the experiences of older adults and challenge the myth that older adults are not vulnerable to sexually transmitted infections (STIs).

## Additional files

Additional file 1: In-depth interview guide. (PDF 191 kb)

Additional file 2: Focus group discussion guide. (PDF 185 kb)

## Data Availability

Data from this study contains information that might compromise the confidentiality and privacy of the participants. However, upon request from the author, some information may be availed. A request can be submitted to chiri2809@gmail.com.

## Abbreviations

AIDS: Acquired Immunodeficiency Syndrome
AMPATH: Academic Model Providing Access to Healthcare
AMRS: AMPATH medical records system
ART: Antiretroviral treatment
CCC: Comprehensive care clinics
CDM: Chronic disease management
FGD: Focus group discussion
HIV: Human immunodeficiency virus
IDI: In depth interview
LTFU: Lost to follow-up
MRC: Medical Research Council
MTRH: Moi Teaching and Referral Hospital
TB: Tuberculosis
UNAIDS: Joint United Nations Programme on HIV/AIDS
UVRI: Uganda Virus Research Institute
WHO: World Health Organization
